# Advisory Committee on Immunization Practices Recommended Immunization Schedule for Adults Aged 19 Years or Older — United States, 2021

**DOI:** 10.15585/mmwr.mm7006a2

**Published:** 2021-02-12

**Authors:** Mark S. Freedman, Kevin Ault, Henry Bernstein

**Affiliations:** ^1^Immunization Services Division, National Center for Immunization and Respiratory Diseases, CDC; ^2^University of Kansas Medical Center, Kansas City, Kansas; ^3^Zucker School of Medicine at Hofstra/Northwell, Hempstead, New York; ^4^Department of Pediatrics, Cohen Children’s Medical Center, New Hyde Park, New York.

At its October 2020 meeting, the Advisory Committee on Immunization Practices (ACIP)* approved the Recommended Immunization Schedule for Adults Aged 19 Years or Older, United States, 2021. After the Emergency Use Authorization of Pfizer-BioNTech COVID-19 vaccine by the Food and Drug Administration, ACIP issued an interim recommendation for use of Pfizer-BioNTech COVID-19 vaccine in persons aged ≥16 years at its December 12, 2020, emergency meeting (*1*). In addition, ACIP approved an amendment to include COVID-19 vaccine recommendations in the child and adolescent and adult immunization schedules. After Emergency Use Authorization of Moderna COVID-19 vaccine by the Food and Drug Administration, ACIP issued an interim recommendation for use of Moderna COVID-19 vaccine in persons aged ≥18 years at its December 19, 2020, emergency meeting (*2*).

The 2021 adult immunization schedule summarizes ACIP recommendations, including several changes from the 2020 immunization schedule^†^ on the cover page, two tables, and accompanying notes found on the CDC immunization schedule website (https://www.cdc.gov/vaccines/schedules). Health care providers are advised to use the tables and the notes together. This adult immunization schedule is recommended by ACIP (https://www.cdc.gov/vaccines/acip) and approved by CDC (https://www.cdc.gov), the American College of Physicians (https://www.acponline.org), the American Academy of Family Physicians (https://www.aafp.org), the American College of Obstetricians and Gynecologists (https://www.acog.org), the American College of Nurse-Midwives (https://www.midwife.org), and the American Academy of Physician Assistants (https://www.aapa.org).

ACIP’s recommendations on use of each vaccine are developed after in-depth reviews of vaccine-related data, including disease epidemiology and societal impacts, vaccine efficacy and effectiveness, vaccine safety, quality of evidence, feasibility of program implementation, and economic analyses of immunization policy (*3*). The adult immunization schedule is published annually to consolidate and summarize updates to ACIP recommendations on vaccination of adults and to assist health care providers in implementing current ACIP recommendations. The use of vaccine trade names in this report and in the adult immunization schedule is for identification purposes only and does not imply endorsement by ACIP or CDC.

For further guidance on the use of each vaccine, including contraindications and precautions, and any updates that might occur between annual updates to the adult immunization schedule, health care providers are referred to the respective ACIP vaccine recommendations at https://www.cdc.gov/vaccines/hcp/acip-recs.^§^ Printable versions of the 2021 adult immunization schedule and ordering instructions are available at https://www.cdc.gov/vaccines/schedules/hcp/adult.html#note.

## Changes in the 2021 Adult Immunization Schedule

Vaccine-specific changes in the 2021 immunization schedules for adults aged ≥19 years include new or updated ACIP recommendations for influenza vaccine (*4*), hepatitis A vaccine (HepA) (*5*), hepatitis B vaccine (HepB) (*6*), human papillomavirus (HPV) vaccine (*7*), pneumococcal vaccines (*8*), meningococcal serogroups A, C, W, and Y (MenACWY) vaccines (*9*), meningococcal B (MenB) vaccines (*9*), and zoster vaccine (*10*).

### Cover page

• The abbreviation for live attenuated influenza vaccine (LAIV) was changed to LAIV4.

• The abbreviation for live recombinant influenza vaccine (RIV) was changed to RIV4.

• MenQuadfi has been added to the list of MenACWY vaccines.

• Abbreviations for the three types of MenACWY vaccines have been added.

• ZVL (zoster vaccine live or Zostavax) has been removed from the table and from the Injury Claims section because the vaccine is no longer available in the U.S. market.

• A link to FAQs for shared clinical decision-making has been added under the Helpful Information section.

### Table 1

• **Tdap row:** This row has been split in half. The upper half is purple to indicate vaccination is recommended for adults with an additional risk factor or another indication (i.e., during each pregnancy and for wound management); the lower half is yellow, indicating vaccination is recommended for adults who meet age requirement, lack documentation of vaccination, or lack evidence of past infection. In addition, text overlay was added to the purple half of the row that states “1 dose Tdap with each pregnancy; 1 dose Td/Tdap for wound management (see notes) for clarification.”

• **MMR row:** The yellow color was extended through age 50–64 years to reflect the age of persons born in or after 1957.

• **VAR row:** The line between the yellow color and the purple color has been shifted to the left to reflect the age of persons born in or after 1980.

**• Zoster row:** Zostavax (ZVL) was deleted because it is no longer available in the U.S. market, and the text “RZV is preferred” was deleted.

• **PCV13 row:** In the column for 65 years and older, the text overlay in the blue box was changed from “≥65 years” to “1 dose.”

### Table 2

• **MMR row**: An asterisk was added after “Not Recommended” to indicate that MMR vaccine should be administered after pregnancy. A line was added between the pregnancy column and the immunocompromised column to separate them.

• **VAR row**: An asterisk was added after “Not Recommended” to indicate that VAR vaccine should be administered after pregnancy. A line was added between the pregnancy column and the immunocompromised column to separate them. In the column for “HIV infection with a CD4 count ≥200 cells/mm^3^,” the color is now blue, indicating that vaccination is recommended, using shared clinical decision-making to reflect that this vaccine recommendation may be considered for this group.

• **Zoster row:** Zostavax (ZVL) has been removed because it is no longer available in the U.S. market. In the pregnancy column, the pink color for “Delay until after Pregnancy” has been replaced with gray because RZV is not recommended during pregnancy.

• **HPV row**: In the pregnancy column, the pink color for “Delay until after Pregnancy” has been replaced with red for “Not Recommended.” This was changed to simplify the schedule because the vaccine is not recommended during pregnancy and should be delayed until after pregnancy. In addition, an asterisk was added after “Not Recommended” to indicate HPV vaccine should be administered after pregnancy. The text overlay spanning the columns “Asplenia, complement deficiencies” through “Men who have sex with men” has been changed to state “2 or 3 doses through age 26 years depending on age at initial vaccination or condition.”

• **HepB row**: The text overlay has been changed to state “2, 3, or 4 doses, depending on vaccine or condition.” In the diabetes column, the box has been split in half. The upper half is yellow and has text overlay “<60 years” to indicate hepatitis B vaccine is routinely recommended for adults aged <60 years with diabetes. The lower half is blue and has text overlay “≥60 years” to indicate shared clinical decision-making should be used for vaccinating persons aged ≥60 years who have diabetes with hepatitis B vaccine.

### Notes

• The notes are presented in alphabetical order. Edits have been made throughout the Notes section to harmonize language between the child/adolescent and the adult immunization schedules to the greatest extent possible.

• **Additional Information:** A section has been added to include language for COVID-19 vaccination recommendations.

• **HepA:** Under “Travel in countries with high or intermediate endemic hepatitis A,” text has been added for the accelerated Twinrix schedule: “HepA-HepB combination vaccine or Twinrix may be administered on an accelerated schedule of 3 doses at 0, 7, and 21–30 days, followed by a booster dose at 12 months.”

• **HepB:** Under “Special Situations,” text has been added to indicate that hepatitis B vaccination for persons aged ≥60 years with diabetes is recommended, using shared clinical decision-making.

• **HPV:** Minor wording changes were made to now read “HPV vaccination recommended for all persons through age 26 years.” Under routine vaccination, the text was reformatted to match the Child/Adolescent schedule and now reads “Age 15 years or older at initial vaccination: 3-dose series at 0, 1–2 months, 6 months (minimum intervals: dose 1 to dose 2: 4 weeks / dose 2 to dose 3: 12 weeks / dose 1 to dose 3: 5 months; repeat dose if administered too soon.” In addition, a bullet was added stating that no additional doses of HPV are recommended after completing a series at the recommended dosing intervals using any HPV vaccine. Under “Shared Clinical Decision-Making,” the text was modified to say “Some adults aged 27–45 years: based on shared clinical decision-making, 2- or 3-dose series as above.” Under “Special situations,” two bullets were added, one stating “Age ranges recommended above for routine and catch-up vaccination or shared clinical decision-making also apply in special situations” and the other stating “Immunocompromising conditions, including HIV infection: 3-dose series as above, regardless of age at initial vaccination.”

• **Influenza vaccination:** In “Special situations,” regarding an “Egg allergy – any symptom other than hives,” this text was added: “If using an influenza vaccine other than RIV4 or ccIIV4 , administer in medical setting under supervision of health care provider who can recognize and manage severe allergic reactions.” Two additional bullets were added: “Severe allergic reactions to any vaccine can occur even in the absence of a history of previous allergic reaction. Therefore, all vaccination providers should be familiar with the office emergency plan and certified in cardiopulmonary resuscitation” and “A previous severe allergic reaction to influenza vaccine is a contraindication to future receipt of the vaccine.” Lastly, an additional bullet about LAIV4 and antivirals was added: “LAIV4 should not be used if influenza antiviral medications oseltamivir or zanamivir was received within the previous 48 hours, peramivir within the previous 5 days, or baloxavir within the previous 17 days.”

• **Meningococcal vaccination:** Under “Special situations for MenACWY,” MenQuadfi (MenACWY-TT) vaccine was added to all relevant sections because it is now licensed. For MenACWY booster doses, text was added to say “Booster dose recommendations for groups listed under ‘Special situations’ and in an outbreak setting (e.g., in community or organizational settings, and among men who have sex with men) and additional meningococcal vaccination information, see https://www.cdc.gov/mmwr/volumes/69/rr/rr6909a1.htm.” For MenB booster doses, text was added to say “Booster dose recommendations for groups listed under ‘Special situations’ and in an outbreak setting (e.g., in community or organizational settings and among men who have sex with men) and additional meningococcal vaccination information, see https://www.cdc.gov/mmwr/volumes/69/rr/rr6909a1.htm.”

• **Pneumococcal vaccination:** The link has been updated for routine vaccination in persons aged ≥65 years (https://www.cdc.gov/mmwr/volumes/68/wr/mm6846a5.htm?s_cid). Under the Shared clinical decision-making section, bullets have been reordered as follows:

o PCV13 and PPSV23 should not be administered during the same visit.

o If both PCV13 and PPSV23 are to be administered, PCV13 should be administered first.

o PCV13 and PPSV23 should be administered at least 1 year apart.

• **Tdap:** The information for wound management has been updated: “Wound management: Persons with 3 or more doses of tetanus toxoid-containing vaccine: For clean and minor wounds, administer Tdap or Td if more than 10 years since last dose of tetanus toxoid-containing vaccine; for all other wounds, administer Tdap or Td if more than 5 years since last dose of tetanus toxoid-containing vaccine. Tdap is preferred for persons who have not previously received Tdap or whose Tdap history is unknown. If a tetanus toxoid-containing vaccine is indicated for a pregnant woman, use Tdap. For detailed information, see https://www.cdc.gov/mmwr/volumes/69/wr/mm6903a5.htm.”

• **Zoster vaccination:** References have been removed to previous receipt of ZVL (zoster vaccine live or Zostavax) dose when considering vaccination of persons aged ≥50 years with RZV (recombinant zoster vaccines or Shingrix) and the bullet about ZVL for persons aged ≥60 years was deleted because ZVL is no longer available in the U.S. market.

## Additional Information

The Recommended Adult Immunization Schedule, United States, 2021, is available at https://www.cdc.gov/vaccines/schedules/hcp/adult.html and in the *Annals of Internal Medicine*. The full ACIP recommendations for each vaccine are also available at https://www.cdc.gov/vaccines/hcp/acip-recs/index.html. All vaccines identified in Tables 1 and 2 (except zoster vaccine) also appear in the Recommended Immunization Schedule for Children and Adolescents, United States, 2021 (https://www.cdc.gov/vaccines/schedules/downloads/child/0-18yrs-child-combined-schedule.pdf). The notes for vaccines that appear in both the adult immunization schedule and the child and adolescent immunization schedule have been harmonized to the greatest extent possible.

## Footnotes

* Recommendations for routine use of vaccines in adults are developed by ACIP, a federal advisory committee chartered to provide expert external advice and guidance to the CDC director on use of vaccines and related agents for the control of vaccine-preventable diseases in the civilian population of the United States. Recommendations for routine use of vaccines in adults are harmonized to the greatest extent possible with recommendations made by the American Academy of Pediatrics (AAP), the American Academy of Family Physicians (AAFP), and the American College of Obstetricians and Gynecologists (ACOG). ACIP recommendations approved by the CDC director become agency guidelines on the date published in the *Morbidity and Mortality Weekly Report* (MMWR). Additional information about ACIP is available at https://www.cdc.gov/vaccines/acip

† Past immunization schedules are available at https://www.cdc.gov/vaccines/schedules/past.html

§ CDC encourages organizations to use syndication as a more reliable method for displaying the most current and accurate immunization schedules on an organization's website rather than copying these schedules to their websites. Use of content syndication requires a one-time step that ensures an organization’s website displays current schedules as soon as they are published or revised; instructions for the syndication code are available on CDC’s website (https://www.cdc.gov/vaccines/schedules/syndicate.html). CDC also offers technical assistance for implementing this form of content syndication (requests can be e-mailed to ncirdwebteam@cdc.gov).
